# Pursuing Clinical Predictors and Biomarkers for Progression in ILD: Analysis of the Pulmonary Fibrosis Foundation (PFF) Registry

**DOI:** 10.1007/s00408-024-00694-2

**Published:** 2024-05-16

**Authors:** Sarah E. Chang, Guiquan Jia, Xia Gao, Courtney Schiffman, Sachin Gupta, Paul Wolters, Margaret Neighbors

**Affiliations:** 1 Genentech, Inc, South San Francisco, CA USA; 2 Department of Medicine, University of California, San Francisco, CA USA

**Keywords:** Interstitial Lung disease (ILD), Idiopathic Pulmonary Fibrosis (IPF), Progressive Pulmonary Fibrosis (PPF), Biomarkers

## Abstract

**Introduction:**

Pulmonary fibrosis is a characteristic of various interstitial lung diseases (ILDs) with differing etiologies. Clinical trials in progressive pulmonary fibrosis (PPF) enroll patients based on previously described clinical criteria for past progression, which include a clinical practice guideline for PPF classification and inclusion criteria from the INBUILD trial. In this study, we compared the ability of past FVC (forced vital capacity) progression and baseline biomarker levels to predict future progression in a cohort of patients from the PFF Patient Registry.

**Methods:**

Biomarkers previously associated with pathobiology and/or progression in pulmonary fibrosis were selected to reflect cellular senescence (telomere length), pulmonary epithelium (SP-D, RAGE), myeloid activation (CXCL13, YKL40, CCL18, OPN) and fibroblast activation (POSTN, COMP, PROC3).

**Results:**

PFF or INBUILD-like clinical criteria was used to separate patients into past progressor and non-past progressor groups, and neither clinical criterion appeared to enrich for patients with greater future lung function decline. All baseline biomarkers measured were differentially expressed in patient groups compared to healthy controls. Baseline levels of SP-D and POSTN showed the highest correlations with FVC slope over one year, though correlations were low.

**Conclusions:**

Our findings provide further evidence that prior decline in lung function may not predict future disease progression for ILD patients, and elevate the need for molecular definitions of a progressive phenotype. Across ILD subtypes, certain shared pathobiologies may be present based on the molecular profile of certain biomarker groups observed. In particular, SP-D may be a common marker of pulmonary injury and future lung function decline across ILDs.

**Supplementary Information:**

The online version contains supplementary material available at 10.1007/s00408-024-00694-2.

## Introduction

Pulmonary fibrosis is a clinical phenotype of various interstitial lung diseases (ILD) with differing etiologies [1]. Clinical trials in pulmonary fibrosis enroll patients based on clinical criteria in the absence of reliable molecular biomarkers, and there is guidance to classify progressive pulmonary fibrosis (PPF) by prior symptomatic, radiological or physiological progression [2]. Studies in pulmonary fibrosis have also enrolled using inclusion criteria based on the INBUILD trial, which showed the effect of nintedanib in patients with progressive fibrosing ILD [3]. However, evidence in idiopathic pulmonary fibrosis (IPF) shows that past progression is not always predictive of future progression [4], and studies in PPF and SSc-ILD have shown heterogenous progression of forced vital capacity (FVC) in patients selected by different clinical criteria. [5, 6] Guidance on clinical enrollment criteria for trials combining IPF and non-IPF ILDs is lacking and prognostic biomarkers to aid in the selection of progressing ILD patients are needed. Moreover, improved molecular phenotyping of ILDs could also inform clinical management and identify new targets for ILD patients.

In this study, we analyzed pulmonary function test data and serum biomarker measurements in a real world cohort of IPF and non-IPF ILD patients from the PFF Patient Registry [7]. Our objectives were to (1) examine how two criteria for past progression in lung function decline performed to predict future progression, and whether biomarker levels differ in the progressor population, (2) compare the levels of pre-specified biomarkers in IPF and non-IPF ILDs, (3) assess if these biomarkers are prognostic of future pulmonary function decline.

## Methods

Characteristics of patients in the registry included mean baseline age of 68.3 years, 69% FVC predicted percent, and 41.7% DLCO predicted percent (Supplementary Table 1). Patients were divided into four ILD diagnosis groups: Idiopathic pulmonary fibrosis (IPF), Idiopathic Interstitial Pneumonia (IIP), Connective Tissue Disease-associated ILDs (CTD-ILD), and Other based on available data and sample sizes (Supplementary Table 2). Vendor procured healthy controls were analyzed alongside patient serum samples (Supplementary Table 3). Pre-specified biomarkers associated with pathobiology and/or progression in pulmonary fibrosis were measured at baseline to reflect cellular senescence (telomere length) [8, 9], pulmonary epithelium (SP-D, RAGE), myeloid activation (CXCL13, YKL40, CCL18, OPN) and fibroblast activation (POSTN, COMP, PROC3) [10–15]. Telomere length was measured using qPCR. ProteinSimple (San Jose, CA) Ella immunoassays were used to measure levels of SPD, RAGE, OPN, CCL18, YKL40 and COMP. Periostin and Pro-C3 levels were measured using Elecsys assays (Roche Diagnostics, Penzberg, Germany).

## Results

**Fig. 1:**
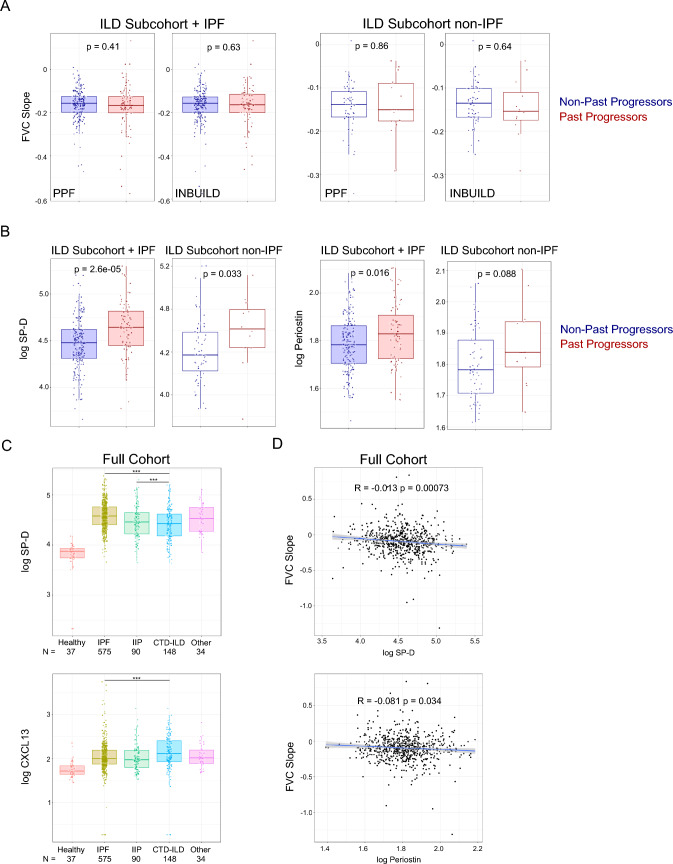
Characterization of disease progression and biomarker profiles across ILD subtypes (A) FVC slope for progressors and non-progressors were compared by unadjusted t-test according to either PPF or INBUILD-like criteria. Left side boxplots show comparisons for the ILD subcohort. Right side boxplots show patients from the subcohort with non-IPF ILDs (*N* = 67) where PPF criteria selected 14 (20.9%) patients and INBUILD criteria selected 12 (17.9%) patients as past progressors. (B) Baseline biomarker levels for Surfactant Protein D (SP-D) and Periostin as compared between progressors and non-progressors by unadjusted *t*-test according to INBUILD-like criteria. Comparisons for both the ILD subcohort and patients with non-IPF ILDs are shown. (C) Comparison of baseline biomarker levels for SP-D and CXCL13 between healthy controls and the full ILD cohort subgrouped by diagnosis using t-test adjusted for multiple comparisons and for age. *p-value < 0.05 **p-value < 0.01 ***p-value < 0.001 ****p < 0.0001. (D) Correlation between baseline biomarker level and FVC slope in the full cohort for SP-D and Periostin with Pearson correlation shown

Patients from the PFF Patient Registry with three years of FVC% data were divided into past progressor and non-past progressor groups based on INBUILD (relative decline in FVC% ≥ 10% in prior two years) or PPF-like (absolute decline in FVC% ≥ 5% and/or absolute decline in DLCO ≥ 10% in prior one year) progression criteria. The full patient cohort was filtered for availability of spirometry data over three years as well as baseline FVC% ≥ 40% and FEV1/FVC ≥ 0.7 based on inclusion criteria for clinical trials in ILD. A subcohort of 277 patients with mixed ILD subtypes, including IPF, was selected to be included in the analysis. PPF-like criteria selected 28.8% of patients (*N* = 80) as past progressors, while the INBUILD-like criteria selected 25.5% of patients (*N* = 71). No difference was observed in future progression of FVC (L) slope, i.e., in the third year, between past progressor and stable patients using either the INBUILD or PPF-like criteria to subset the patients (Fig. 1A). This was true for both the mixed ILD subcohort and the non-IPF ILD subcohort (Fig. 1A). Serum biomarkers levels were compared in patients chosen as past progressors in the mixed ILD subcohort and the non-IPF subcohort. Of the biomarkers tested, only SP-D and POSTN levels were higher in the INBUILD progressor population using both cohorts (Fig. 1B). No biomarker differences were observed in patients designated as progressors using the PPF-like criteria (data not shown).

Baseline biomarker levels were compared between vendor procured healthy controls and the ILD diagnosis groups within the full cohort. All biomarkers measured were differentially expressed in patient groups compared to healthy controls. Most biomarkers levels were comparable between the four ILD diagnosis groups. SP-D levels appeared to be higher in IPF patients compared to IIP and CTD-ILD patients, while CXCL13 levels were higher in CTD-ILD patients compared to IPF patients (Fig. 1C). Correlations between all measured biomarkers were assessed for each ILD diagnosis group. Similar groups of biomarkers related to fibroblast activation or myeloid activation were positively correlated in both IPF and CTD-ILD patient groups (Supplementary Figure 1) which may indicate shared pathobiology. In IIP patients, however, only the myeloid-related proteins were positively correlated (Supplementary Figure 1).

In order to identify molecular targets prognostic of future FVC decline, association between baseline biomarker levels and FVC slope over the subsequent first year was assessed. In the full mixed ILD cohort, SP-D and POSTN levels at baseline showed the highest correlations with FVC slope, though correlations were low (Fig. 1D). The association between SP-D and FVC slope remained significant when adjusted for baseline patient characteristics (Supplementary Table 4). When divided by ILD diagnosis groups, SP-D correlated with FVC slope only in IPF and IIP patients while POSTN correlated with FVC slope only in IPF patients (data not shown). Apparent differences seen in time to ≥ 10% decline in FVC%, death, or respiratory hospitalization for patients with higher levels of SP-D appeared to be transient (Supplementary Figure 2). This was also true for patients with lower levels of sRAGE and increased levels of CXCL13 (Supplementary Figure 2). In an analysis of biomarker levels in progressors vs non-progressors, SP-D, OPN, CCL18, POSTN, CXCL13, and COMP levels were higher in the progressor population while sRAGE levels were lower in the progressor population (Supplementary Figure 3). However, the broad overlap of biomarker levels in progressors vs non-progressors indicates that these markers may not reliably identify progressors in clinical application.

## Discussion

Limitations of our analysis include missing data and small patient subpopulations for non-IPF ILD diagnosis subgroups. As a result, in our criteria analysis, guidelines based on non-IPF ILDs were applied to a majority IPF patient population. While both criteria included radiological symptoms in their definition of prior progression, the number of HRCT measurements in our cohort did not allow for this data to be included in our analysis. Our analysis is also limited to a pre-specified set of proteins, which include only some of the previously studied biomarkers in IPF and other ILDs, and did not include a replication cohort.

Our findings provide further evidence that prior decline in lung function (using either INBUILD or PPF-like criteria) may not predict future disease progression for trials enrolling both IPF and non-IPF ILDs, and elevates the need for molecular definitions of patients with a progressive phenotype. Comparison of the molecular profiles between patients with IPF and other non-IPF ILDs for the biomarkers assessed in this study points to certain shared pathobiologies that help inform which interventions may have potential to benefit mixed ILD populations. In particular, SP-D may be a common marker of pulmonary injury and future lung function decline across some ILDs.

### Supplementary Information

Below is the link to the electronic supplementary material.

Supplementary file1 (PDF 1164 KB)
