# Supplementary material for: Pursuing Clinical Predictors and Biomarkers for Progression in ILD: Analysis of the Pulmonary Fibrosis Foundation (PFF) Registry
Source: Lung. 2024 May 16;202(3):269–73. doi: 10.1007/s00408-024-00694-2 (PMC11142961; doi:10.1007/s00408-024-00694-2)
Supplement: Supplementary file 1 — Supplementary file1 (PDF 1164 KB) [file 408_2024_694_MOESM1_ESM.pdf]

Supplementary Table 1

Full Cohort<br>Baseline Characteristic	IPF<br>(N=575)	IIP<br>(N=90)	CTD-ILD<br>(N=148)	Other<br>(N=34)	Total<br>(N=847)
Baseline Age					
n	575	90	148	34	847
Mean (SD)	71.0 (8.0)	65.1 (10.8)	61.3 (12.4)	62.7 (10.7)	68.3 (10.2)
Median	71.3	67.3	62.7	62.1	69.8
Min-max	23.0-91.9	33.8-85.2	19.8-86.6	44.9-84.9	19.8-91.9
Sex					
Female	156 (27.1%)	29 (32.3%)	94 (63.5%)	17 (50%)	296 (35.0%)
Male	419 (72.9%)	61 (67.8%)	54 (36.5%)	17 (50%)	551 (65.0%)
Race/ethnicity					
White	544 (94.6%)	80 (88.9%)	115 (77.7%)	30 (88.2%)	769 (90.8%)
Nonwhite	31 (5.4%)	10 (11.1%)	33 (22.3%)	4 (11.8%)	78 (9.2%)
BMI					
n	533	84	138	34	789
Mean (SD)	29.19 (5.4)	30.9 (7.3)	29.8 (7.5)	28.0 (6.2)	29.4 (6.1)
Median	28.5	30.1	28.7	28.2	28.7
Min-max	15.3-59.4	12.8-58.3	12.1-63.3	17.2-39.9	12.1-63.3
Tobacco Use History					
Current	2 (0.003%)	4 (4.4%)	3 (2.0%)	2 (5.9%)	11 (1.3%)
Former	358 (62.2%)	42 (46.7%)	63 (42.6%)	16 (47.1%)	479 (56.6%)
Never	415 (72.2%)	44 (48.9%)	82 (55.4%)	16 (47.1%)	357 (42.1%)
Time from IPF diagnosis to baseline (days)					
n	575	90	148	34	847
Mean (SD)	942.3 (982)	1148.7 (1369)	1315.8 (1305)	1438.6 (1334)	1049.4 (1114)
Median	622	590.5	858.5	948.5	680
Min-max	-3-6712	-8-6332	0-5694	26-5166	-8-6712
Standard of Care at Enrollment					
Pirfenidone	221 (38.4%)	5 (5.6%)	4 (2.7%)	4 (12.5%)	234 (27.6%)
Nintedanib	172 (29.9%)	4 (4.4%)	0	1 (3.1%)	177 (20.9%)
Neither	182 (31.7%)	81 (90%)	144 (97.3%)	27 (84.4%)	436 (51.5%)
Baseline %FVC					
n	537	84	138	34	793
Mean (SD)	68.5 (16.8)	72.2 (20.5)	68.7 (19.1)	71.1 (20.6)	69.0 (17.8)
Median	67.8	70.0	65.6	67.1	67.5
Min-max	27.4-120.4	26.8-117.5	29.3-120.2	25.4-117.2	25.4-120.4
<50%	74 (13.8%)	9 (10.7%)	22 (15.9%)	3 (8.8%)	108 (13.6%)
50-80%	328 (61.1%)	47 (60.0%)	82 (59.4%)	22 (64.7%)	479 (60.4%)
≥80%	135 (25.1%)	28 (33.3%)	34 (24.6%)	9 (26.5%)	206 (25.9%)
Baseline FEV1/FVC					
n	537	84	138	34	793
Mean (SD)	0.82 (0.08)	0.81 (0.11)	0.80 (0.09)	0.79 (0.08)	0.8 (0.1)
Median	0.82	0.80	0.81	0.79	0.8
Min-max	0.45-1.56	0.51-1.32	0.52-1.26	0.58-0.95	0.4-1.6
Baseline Hgb-corrected DLCO (% predicted)					
n	215	45	58	14	332

Mean (SD)<br>Median<br>Min-max	39.8 (14.2)<br>38.7<br>9.8-93.3	46.2 (20.1)<br>43.1<br>14.8-98.4	46.1 (19.1)<br>43.1<br>10.4-96.6	38.6 (19.2)<br>39.2<br>7.7-75.3	41.7 (16.4)<br>40.0<br>7.7-98.4
FVC Slope 1 Year (L/year)<br>n<br>Mean (SD)<br>Median<br>Min-max	 469<br>-0.13 (0.45)<br>-0.11<br>-2.17-1.99	 70<br>-0.04 (0.43)<br>-0.07<br>-1.22-1.22	 111<br>-0.06 (0.36)<br>-0.07<br>-1.02-1.86	 28<br>0.11.2 (0.55)<br>-0.05<br>-0.47-2.23	 688<br>-0.10 (0.44)<br>-0.09<br>-2.17-2.23
FVC Slope 2 Years (L/year)<br>n<br>Mean (SD)<br>Median<br>Min-max	 485<br>-0.16 (0.37)<br>-0.13<br>-2.17-1.65	 75<br>-0.09 (0.34)<br>-0.08<br>-1.22-0.97	 120<br>-0.03 (0.28)<br>-0.03<br>-0.75-1.86	 29<br>0.02 (0.34)<br>-0.02<br>-0.63-1.09	 719<br>-0.12 (0.35)<br>-0.09<br>-2.17-1.86
Corticosteroids*<br>Yes<br>No	 189 (32.9%)<br>386 (67.1%)	 50 (55.6%)<br>40 (44.4%)	 105 (70.9%)<br>43 (29.1%)	 27 (79.4%)<br>7 (20.6%)	 371 (43.8%)<br>476 (56.2%)
Immunosuppressants**<br>Yes<br>No	 41 (7.1%)<br>534 (92.9%)	 42 (46.7%)<br>48 (53.3%)	 134 (90.5%)<br>14 (9.5%)	 13 (38.2%)<br>21 (61.8%)	 230 (27.2%)<br>617 (72.8%)

*Conmed Group: Corticosteroids at any time (to treat lung condition) (Do not include topical treatments)

** Conmed Groups: Mycophenolate (CellCept, etc.), Hydroxychloroquine (Plaquenil, etc.), Rituximab (Rituxan, etc.), Azathioprine (Imuran, etc.), Tacrolimus (Prograf, etc.), Cyclosporin A (Cyclosporine modified, Gengraf, Neoral, Restasis, Sandimmune, Sandoz Cyclosporine, etc.), Adalimumab (Humira, etc.), Etanercept (Enbrel, etc.), Tocilizumab (Actemra, etc.), Infliximab (Remicade, etc.), Cyclophosphamide (Cytoxan, etc.), Methotrexate (Trexall, etc.), Abatacept (Orencia, etc.), Golimumab (Simponi, etc.), Leflunomide (Arava, etc.), Belimumab (Benlysta, LymphoStat-B, etc.); at any time

Supplementary Table 2

**Diagnosis Subgroup**	
IPF (N=575)	"IIP: Idiopathic pulmonary fibrosis"
IIP (N=90)	"IIP: Unclassifiable"<br>"IIP: Nonspecific interstitial pneumonia (NSIP) - Fibrotic"<br>"IIP: Lymphocytic interstitial pneumonia (LIP)"<br>"IIP: Desquamative interstitial pneumonitis (DIP)"<br>"IIP: Nonspecific interstitial pneumonia (NSIP) - Cellular"<br>"IIP: Cryptogenic organizing pneumonitia (COP)"<br>"IIP: Pleuroparenchymal fibroelastosis (PPFE)"
CTD-ILD (N=148)	"Collagen Vascular Disease/Autoimmune Diseases: Rheumatoid arthritis"<br>"Collagen Vascular Disease/Autoimmune Diseases: Antisynthetase syndrome" "Collagen Vascular Disease/Autoimmune Diseases: Systemic sclerosis/scleroderma"<br>"Collagen Vascular Disease/Autoimmune Diseases: Sjogrens syndrome"<br>"Collagen Vascular Disease/Autoimmune Diseases: Polymyositis/Dermatomyositis"<br>"Collagen Vascular Disease/Autoimmune Diseases: Systemic lupus erythematosus"<br>"Collagen Vascular Disease/Autoimmune Diseases: Undifferentiated connective tissue disease"<br>"Collagen Vascular Disease/Autoimmune Diseases: Mixed connective tissue disease"<br>"Collagen Vascular Disease/Autoimmune Diseases: ANCA - vasculitis/pulmonary capilaritis/vasculitis"<br>"Collagen Vascular Disease/Autoimmune Diseases: Ankylosing spondylitis" "Collagen Vascular Disease/Autoimmune Diseases: Granulomatosis with polyangiitis"
Other (N=34)	"Hypersensitivity pneumonitis: Other (specify)”<br>"Occupational exposure: Asbestosis"<br>"Occupational exposure: Berylliosis"<br>"Occupational exposure: Hard metal" "Occupational exposure: Other (specify)"<br>"Other specific ILD: Chronic aspiration"<br>"Other specific ILD: Pulmonary Langerhans cell histiocytosis (Eosinophilic granuloma)"<br>"Other specific ILD: Other (specify)"<br>"Drug or Treatment Induced: Radiation"<br>"Drug or Treatment Induced: Nitrofurantoin (Macrobid / Macrodantin)"<br>"Drug or Treatment Induced: Other (specify)"

Supplementary Table 3

Baseline Characteristic	Healthy Controls (N=30)
Baseline Age	
n	30
Mean (SD)	66.9 (6.0)
Median	65
Min-max	60-82
Sex	
Female	6 (20%)
Male	24 (80%)
Race/ethnicity	
White	27 (90%)
Nonwhite	3 (10%)

Supplementary Table 4:
Associations of Baseline Biomarkers with FVC Slope. SP-D or Periostin levels were used in a linear regression analysis to predict FVC slope over one year, with and without adjustment for baseline patient characteristics.

	**SP-D FVC Slope over 1 year**		**Periostin FVC Slope over 1 year**	
Covariates Used for Adjustment	Adjusted R^2	p-value	Adjusted R^2	p-value
None	0.01506	0.000733	0.005066	0.0345
Baseline FVC	0.02042	0.0004382	0.009228	0.01802
Age	0.01363	0.00352	0.003638	0.1061
Sex	0.01377	0.003189	0.00374	0.1025
Height	0.01363	0.003354	0.003996	0.09385

Supplementary Figure 1:
Subsets of Biomarkers Correlated Across ILD Diagnosis Groups

IPF

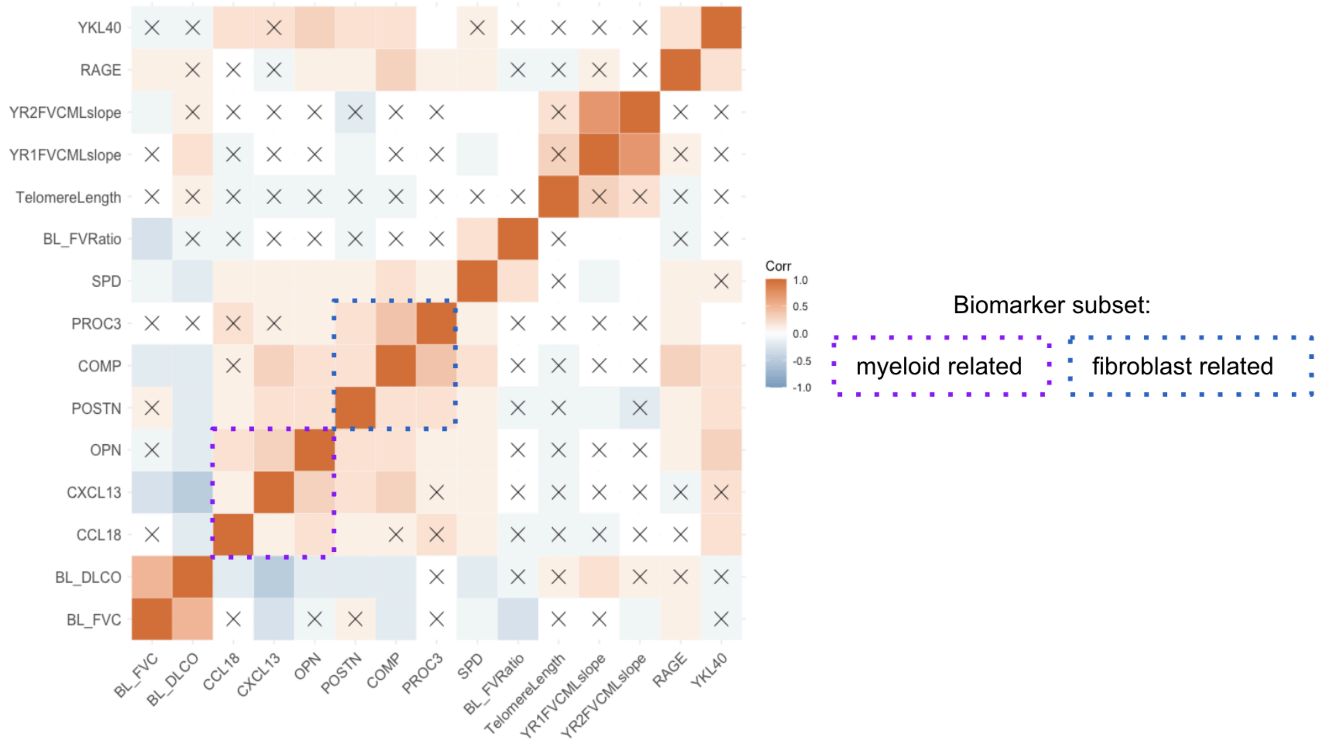

CTD-ILD

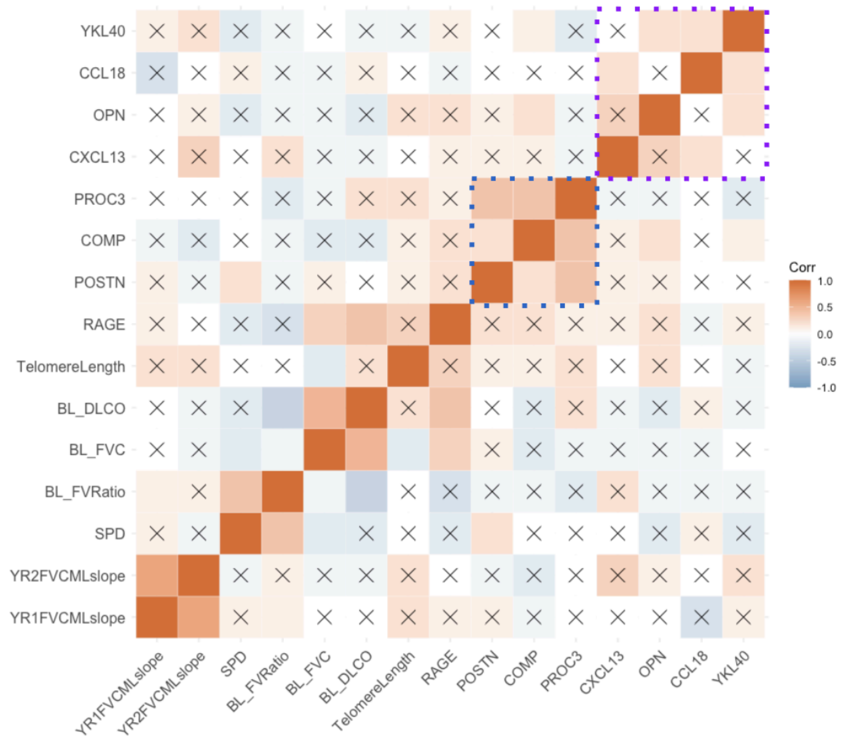
YKL40
CCL18
OPN
CXCL13
PROC3
COMP
POSTN
RAGE
TelomereLength
BL_DLCO
BL_FVC
BL_FVRatio
SPD
YR2FVCMLslope
YR1FVCMLslope
YR1FVCMLslope
YR2FVCMLslope
SPD
BL_FVRatio
BL_FVC
BL_DLCO
TelomereLength
RAGE
POSTN
COMP
PROC3
CXCL13
OPN
CCL18
YKL40
Corr
1.0
0.5
0.0
-0.5
-1.0

IIP

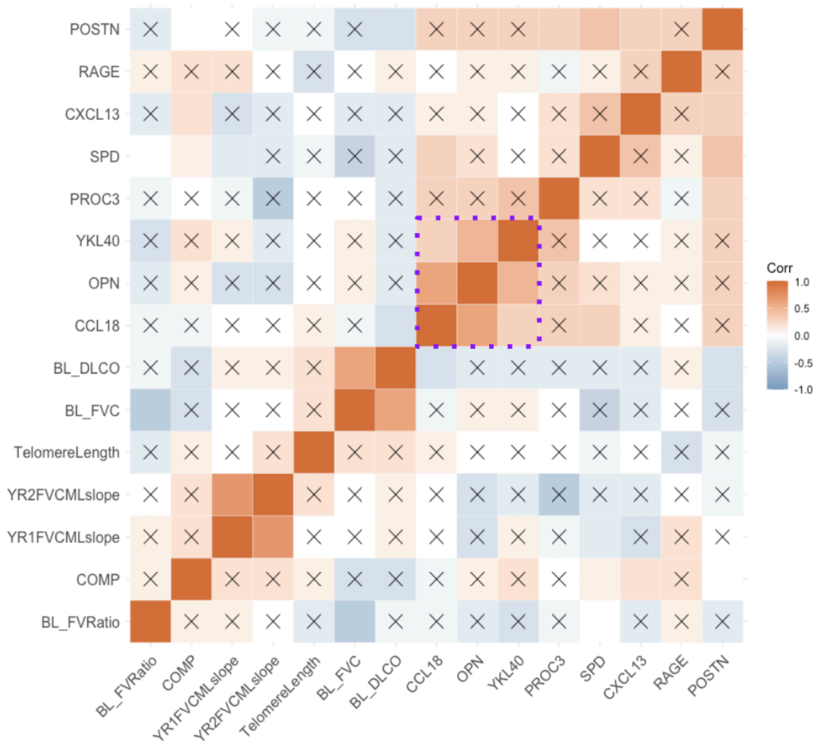
POSTN
RAGE
CXCL13
SPD
PROC3
YKL40
OPN
CCL18
BL_DLCO
BL_FVC
TelomereLength
YR2FVCMLslope
YR1FVCMLslope
COMP
BL_FVRatio
BL_FVRatio
COMP
YR1FVCMLslope
YR2FVCMLslope
TelomereLength
BL_FVC
BL_DLCO
CCL18
OPN
YKL40
PROC3
SPD
CXCL13
RAGE
POSTN
Corr
1.0
0.5
0.0
-0.5
-1.0

Other

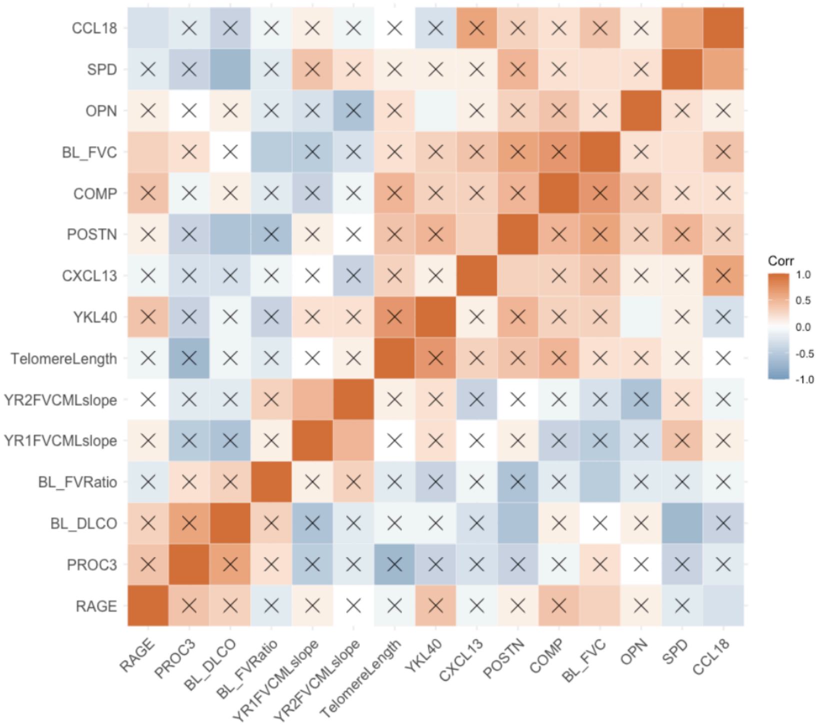

Spearman correlations shown
“X” indicates non-significant p-value

Supplementary Figure 2
Time to Relative Decline in FVC% predicted ≥ 10%, death, or respiratory hospitalization - Stratified by Biomarker Level

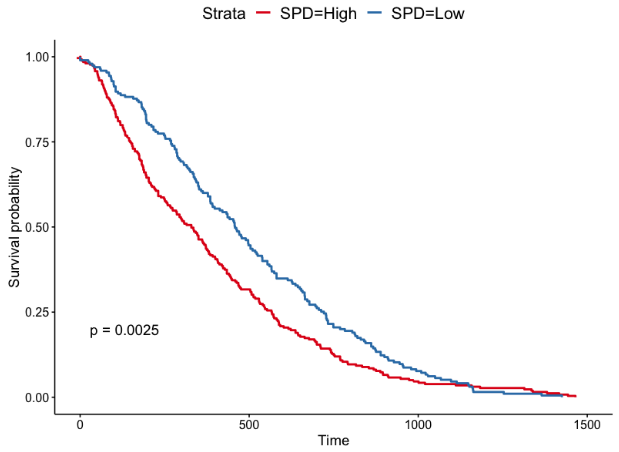

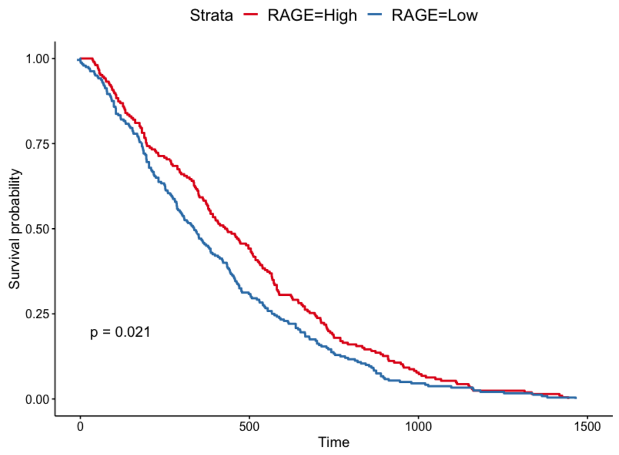

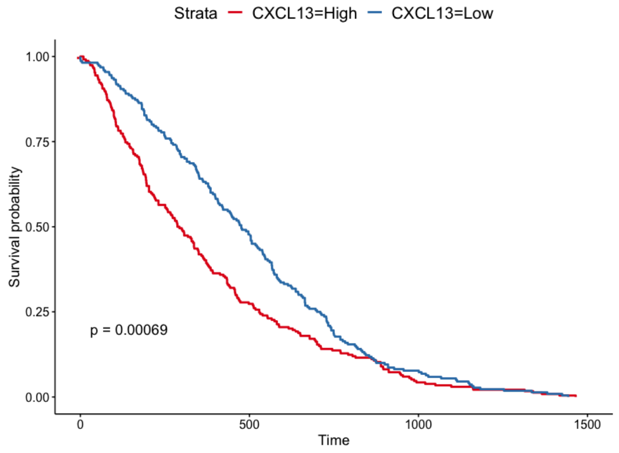

Patients were stratified by median biomarker level into High and Low groups. Time to endpoint analysis was done using relative decline in FVC%, death, or respiratory hospitalization. Time is shown in days on the x-axis. Regular log-rank test was used to determine p-values.

Supplementary Figure 3

Biomarker Levels in Progressors/Non-progressors

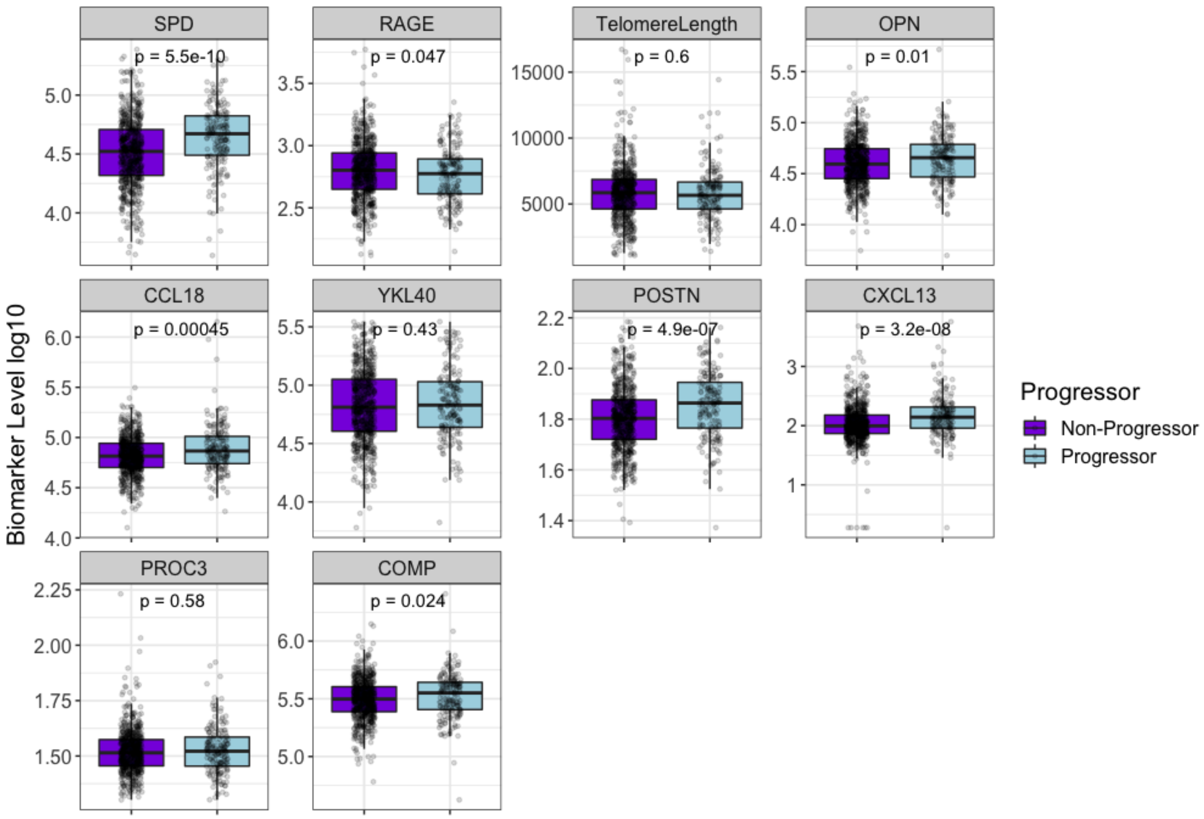

The full patient cohort was evaluated for progression over one year. Progression was defined by FVC% decline ≥ 10%, death, or lung transplant. 186 out of 847 (22%) patients were selected as progressors using this criteria. Differences in biomarker level were compared using unadjusted t-tests.
